# Physicochemical Properties of Fine and Coarse Fly Ash Aerosol Particles from Waste Incineration

**DOI:** 10.1021/acsenvironau.6c00018

**Published:** 2026-06-02

**Authors:** Fanny Bergman, Jenny Rissler, Sara Janhäll, Michael Strand, Edvin Elmroth, Karin Karlfeldt Fedje

**Affiliations:** † Ergonomics and Aerosol Technology, 5193 Lund University, Lund 22100, Sweden; ‡ NanoLund, Lund University, Lund 22100, Sweden; § 388792 RISE Research Institutes of Sweden, Borås 501 15, Sweden; ∥ Built Environment and Energy Technology, Linnaeus University, Växjö 35195, Sweden; ⊥ NG Nordic AB, Kumla 69292, Sweden; # Renova Recycling and Waste Management, Renova AB, Gothenburg 40122, Sweden; ∇ Architecture and Civil Engineering, Chalmers University of Technology, Gothenburg 41296, Sweden

**Keywords:** WtE, circularity, waste incineration, aerosol, elemental size partitioning, sizing

## Abstract

Understanding the physicochemical properties of fly ash from industrial and municipal solid waste incineration is essential for its safe and efficient utilization. This study investigated the particle size distribution and elemental size partitioning in fly ash aerosols from a waste-to-energy (WtE) facility with grate-fired boilers, along with the composition of boiler deposits and ash collected in the electrostatic precipitator (ESP). The physicochemical properties of fly ash were investigated using online aerosol instruments combined with size-selective collection (low-pressure impactor and cyclone-filter setup) for gravimetric and elemental analyses. The particle size distribution was multimodal, with a distinct mass peak at 0.5 μm well separated from two overlapping modes at 30 and 200 μm. Fine particles (<1 μm) represented 16% of the total mass but were dominant in number. Elemental analysis showed that fine particles mainly consisted of Cl, Na, K, Zn, and S. Fine particles were enriched in potentially toxic yet valuable metals (Zn, Cd, Cu, Sb, Pb, Sn). Coarse particles (>1 μm) were dominated by Ca, Si, and Al, while the above-mentioned metals were depleted. Boiler ash resembled the coarse fraction, and ESP ash was a mix of both fine and coarse particles. Increased knowledge of the elemental composition in different size fractions may enable large-scale size separation for improved resource recovery and safer utilization. The composition of the fine particles support targeted salt or metal recovery, while coarse particles are suitable for construction applications.

## Graphical abstract

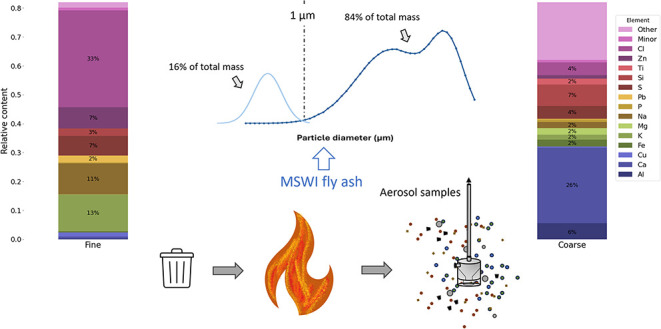

## 1 Introduction

Waste-to-energy (WtE) facilities incinerate nonrecyclable municipal solid waste (MSW) to minimize waste volume and utilize the energy stored in the materials. The ash residue remaining after incineration can be broadly divided into bottom ash and fly ash. Bottom ash is the noncombustible residue at the bottom of the furnace, while ash suspended in the flue gas stream is referred to as fly ash. Modern WtE incinerators are equipped with air pollution control systems that capture fly ash particles,^1^ thereby effectively reducing the release of pollution into the air. The collected ash is typically rich in metals and salts^2,3^ and remains challenging to handle and utilize due to its potentially toxic properties. According to Eurostat, an average of approximately 500 kg of MSW per capita is generated per year in the EU, with 25% of the MSW being treated by incineration. In Sweden, WTE facilities treat as much as 55% of MSW.^4^

In a circular economy, metal-enriched fly ash is a resource with potential for material recycling and secondary use. By treating fly ash, it is possible not only to recover valuable metals but also to utilize the ash in construction.^3^ Full-scale industrial processes have been developed for the recovery of Zn, Cu, Cd, and Pb from WtE fly ash.^5–7^ Improving the extraction efficiency and enhancing the product purity in recovery processes requires a more detailed understanding of the physicochemical properties of fly ash.

Grate-fired WtE incinerators typically operate at high temperatures (following legislation^8^ requiring that flue gases are held at least 850 °C for 2 s) and with a lean air–fuel ratio, resulting in efficient combustion and elimination of toxic organic compounds.^9^ During high-temperature combustion, volatile compounds evaporate, causing enrichment of Cl, S, K, and Na, as well as metals like Zn, Pb, As, and Cd, in the fly ash.^10,11^ During the cooling of the flue gas, nucleation and condensational growth typically result in submicrometer particles, where the fine (<1 μm) size fraction of fly ash is expected to contain high concentrations of volatile species. In addition, fly ash often includes coarse particles (>1 μm) composed of refractory species entrained from the combustion bed and transported with the flue gas.^12,13^ Although a distinct difference in the chemical composition between fine and coarse fly ash particles from WtE incineration has been previously suggested,^12–15^ it has not yet been investigated in detail.

Fly ash formation via either condensation or entrainment suggests that fine- and coarse-mode particles have different elemental compositions and that the chemical forms of the elements may differ between particles of different sizes. Therefore, size-selective filtration approaches can be applied to facilitate the optimized utilization of WtE fly ash. This requires further studies on the physicochemical properties of fly ash. Detailed information on the elemental content and mass concentration in different size fractions of fly ash can only be achieved by sampling directly in the flue gas channel, as submicrometer ash particles collected from electrostatic precipitators (ESPs) are agglomerated, with strong surface forces preventing full dispersion.^16^

Here, we present the physicochemical properties of fly ash particles sampled in-flight from the flue gas channel of a full-scale grate-fired WtE incinerator. We combined online instruments for size distribution measurements with impactor collection and separation of coarse and fine-mode particles using a setup with a cyclone and a filter coupled in series. We also collected and analyzed ash samples from the ESP as well as boiler deposits. The collected ash samples were assessed gravimetrically and analyzed for elemental content. The sampling setup allowed the analysis of the hypothesized nucleation/condensation and entrained fractions of fly ash particles.

## 2 Methods

### 2.1 Incineration Facility

The incineration plant consists of multiple units for grate-fired combustion of MSW, and sampling was performed in two different units. Prior to combustion, the waste is mixed in a storage bunker and subsequently fed to the furnace (Figure 1). In the furnace, primary air is supplied beneath the fuel bed, and secondary air is injected above it. This air-staging strategy promotes efficient combustion by ensuring adequate mixing, maintaining the flue gas residence time, and reducing the emissions of partially combusted carbonaceous compounds.

**Figure 1:**
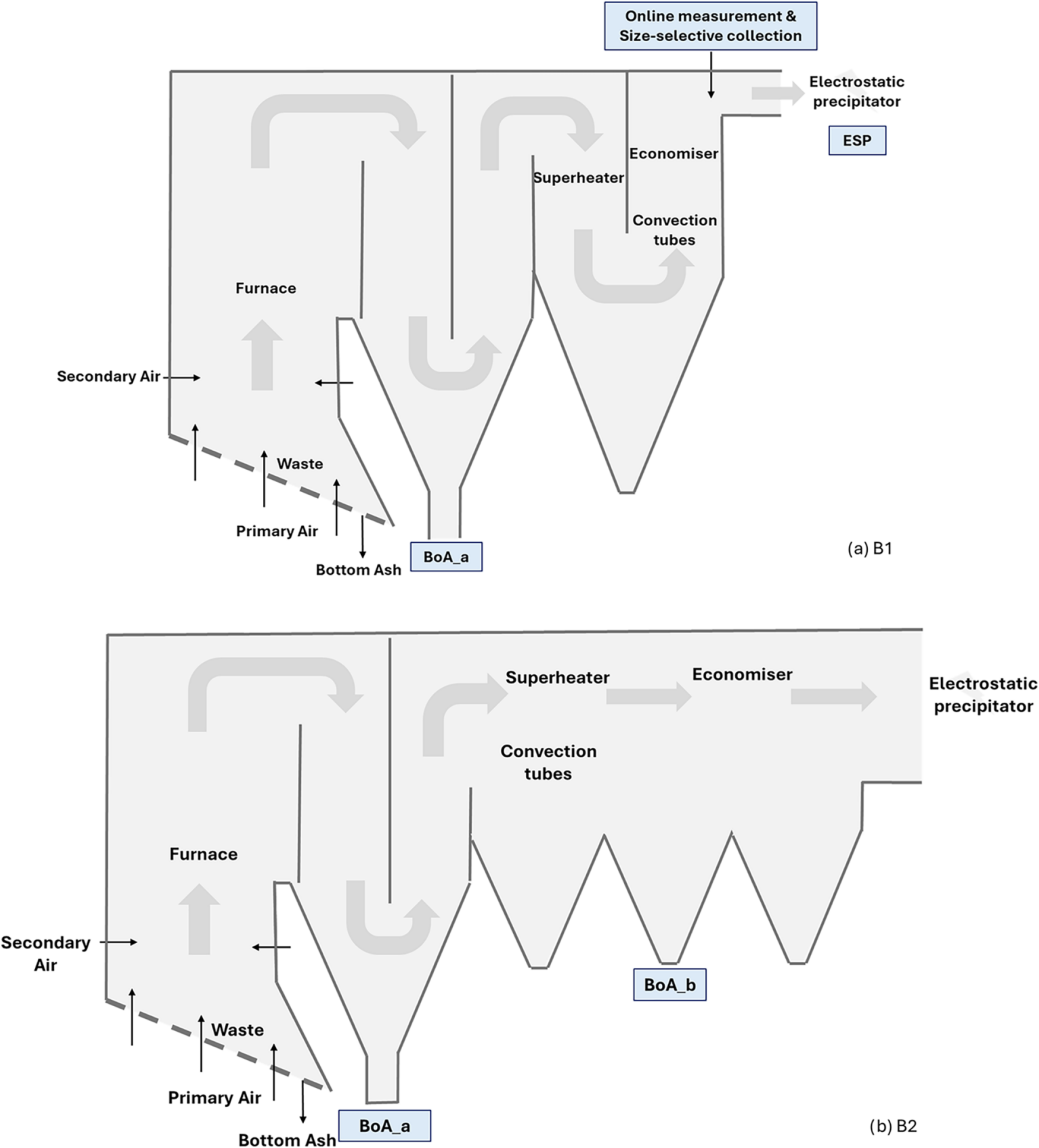
Schematic representation of two different grate-fired boiler designs. The sampling positions are indicated by blue boxes. (a) WtE unit one (B1). (b) WtE unit two (B2).

Most samples were collected from one WtE unit, here referred to as B1 (Figure 1a), but boiler ash was also collected from a second WtE unit, B2 (Figure 1b). Both units have a shared waste bunker. In the incineration process the flue gases are held at least 850 °C for >2 s, fulfilling the European legislation^8^ and ensuring efficient incineration. The boiler thermal capacity is 45 and 54 MW for B1 and B2, with capacities of 16 and 22 t MSW/h, respectively.

Further differences between the WtE units are the different boiler sections. In unit B1, the convection zone is vertical, resulting in two chutes. In unit B2, the convection zone is horizontally aligned with three boiler chutes. Both units have two passes after the secondary combustion chamber. Ash samples from the units were collected from the chute at the bottom of the first and second passes (BoA_a in Figure 1), and in B2 samples, were also collected from the boiler chute (BoA_b in Figure 1b). For B1, sampling from the boiler chute was not possible. Both B1 and B2 have modern flue gas cleaning systems, but as all sampling was performed prior to the flue gas cleaning steps, no further description is included here.

The same facility was included in an earlier study investigating fly ash collected from the ESP, in which it was demonstrated that the elemental composition of the fly ash is comparable to that of other Scandinavian WtE facilities with grate-fired boilers.^14^

#### 2.1.1 Monitoring Boiler Operation during Sampling

To ensure that sampling was conducted under stable operating conditions, the key indicators of proper combustion and plant performance were assessed. These indicator measurements included the flue gas temperature (in the vicinity of the aerosol sampling point) and flue gas flow rate. Temperature and flow rate measurements were performed continuously as part of routine plant monitoring. Figure 2 illustrates the frequency distributions of the indicators over the ten-day periods preceding and following each of the two sampling events (see Section 2.2), along with the average values during the periods when aerosol samples (described in Section 2.2) were collected. Together, these data demonstrate that sampling was conducted during representative and steady plant operations.

**Figure 2:**
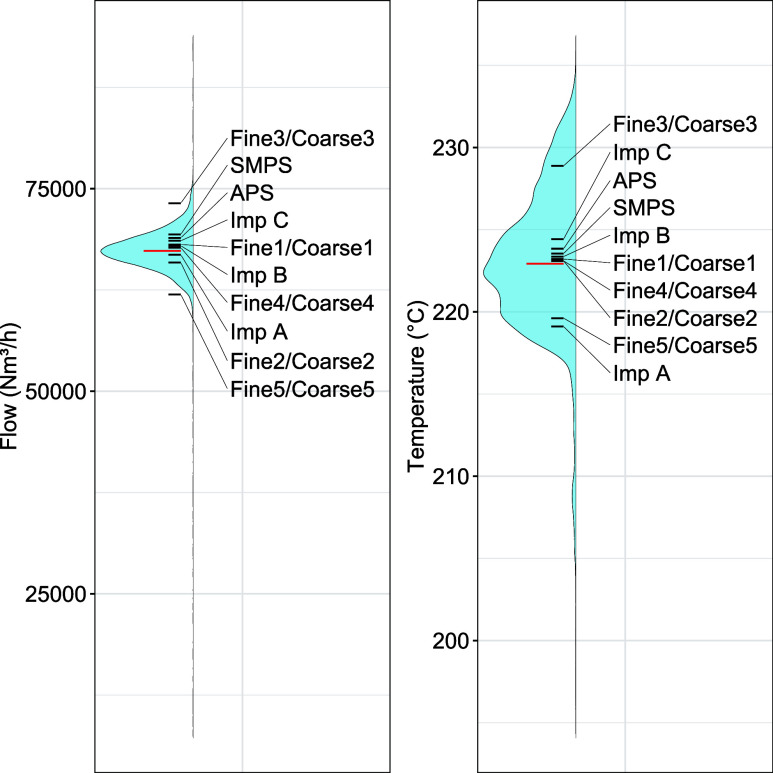
Violin plots indicating the frequency distributions of the flow rate (left panel) and temperature in the vicinity of the aerosol sampling point (right panel) at unit B1. The violin plots contain continuous data 7–10 days before and after the respective campaigns, where the red line corresponds to the median. The measured flow rate and temperature during the collection of the aerosol samples (fine/coarse-sized particles, impactor sampling (Imp), online instruments: aerodynamic particle sizer (APS) and scanning mobility particle sizer (SMPS)) are marked in the plot with the corresponding sample name (see Section 2.2).

### 2.2 Fly Ash Sample Collection

Sampling was conducted on two occasions (January and March) in the same year (2024). On the first occasion, online instrument data were collected (instruments are described in Section 2.3) along with impactor samples, ESP ash, and boiler ash. One set of cyclone-filter samples (setup described below) was collected during the first occasion (Fine1 and Coarse1), while all other cyclone–filter samples were collected during the second occasion. All aerosol samples were collected at B1, as indicated in Figure 1a.

#### 2.2.1 Aerosol Sampling of Coarse- and Fine-Mode Particles (Cyclone–Filter Setup)

Coarse- and fine-mode particle separation was achieved using a cyclone, which collected the coarse particles, coupled in series with a filter holder that collected the finer particles that passed through the cyclone (Figure 3a). A sharp cutoff cyclone with a body diameter of 35 mm was used, which was inserted into the flue gas channel with the inlet facing the flow direction. The cyclone inlet was modified to achieve close to isokinetic conditions. Downstream of the cyclone, a filter holder (FH 90-N, METLAB, Enköping, Sweden) was connected to the cyclone via a 1.6 m long straight tube (inner diameter: 12 mm) and a soft circular 180° bend. The filter holder was equipped with a thermoelement and heater, maintaining the sampling filter at a temperature of ∼90 ± 10 °C to avoid water condensation. Both quartz (MK360, Ahlstrom AB, Jönköping, Sweden) and polycarbonate (Whatman Nuclepore 0.2 μm pore size, Fisher Scientific, Lund, Sweden) filters were used. Aerosol was sampled at a volume flow rate of ∼36 ± 3 l/min, corresponding to a cyclone cutoff at 1.1 ± 0.1 μm (Figure S1). The submicrometer ash particles collected on the filter are referred to as the fine fraction or, equivalently, the fine mode. The collection time for this setup was typically 10–20 min. The collected samples are denoted Fine X and Coarse X, where X denotes the sample occasion with running numbers from 1 to 5.

**Figure 3:**
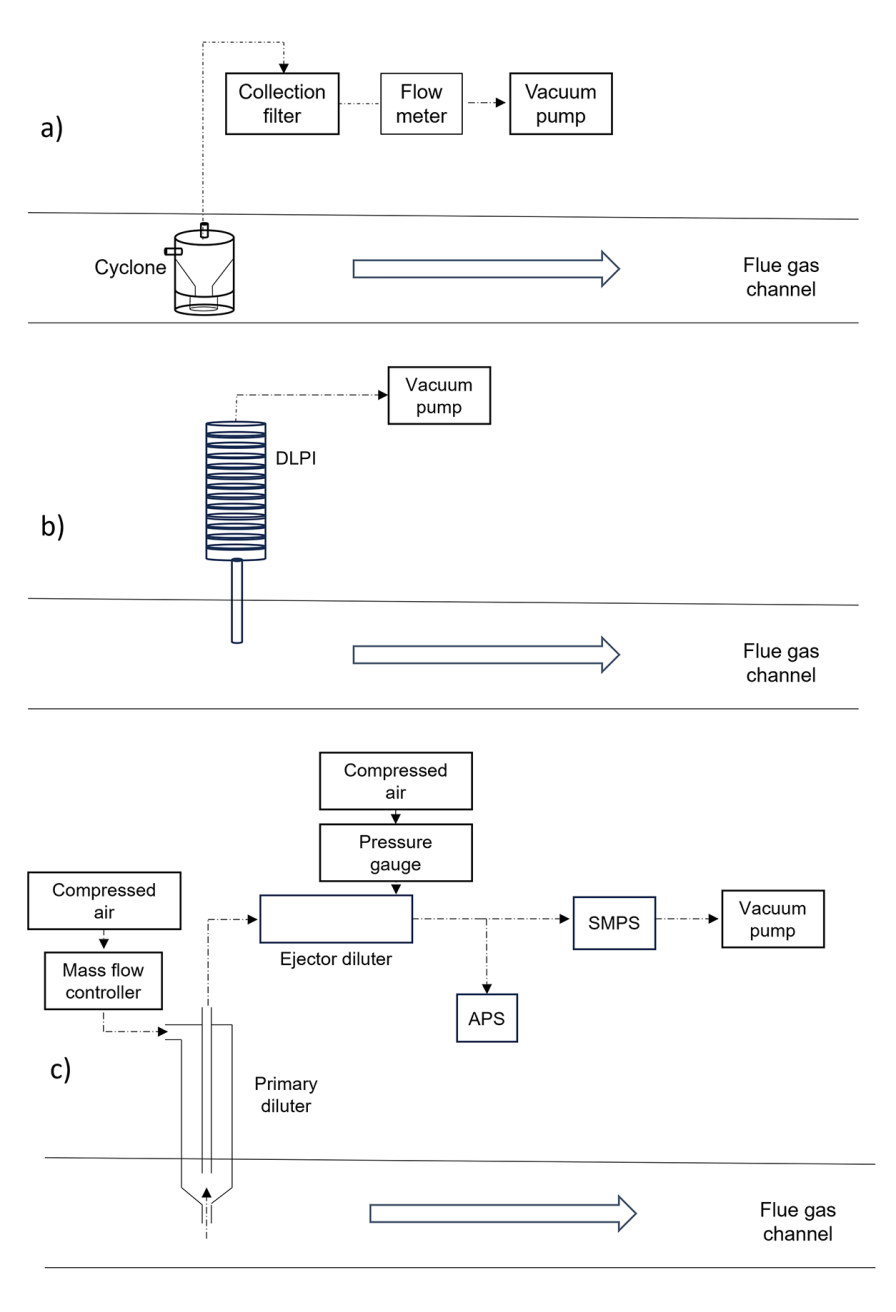
Sampling setup for aerosol sampling, directly from the flue gas channel of the grate-fired WtE boiler. (a) Setup for cyclone and filter sampling, separating coarse and fine particles. (b) Size-selective low-pressure impactor sampling. (c) Online measurements with dilution in two steps.

The total aerosol particle mass concentration was determined from the cyclone–filter sampling as it was performed under close to isokinetic conditions by summing the collected masses of fine and coarse-mode particles.

#### 2.2.2 Impactor Aerosol Sampling

A low-pressure cascade impactor (DLPI, Dekati, Tampere, Finland) was used for size-segregated sampling of fly ash (Figure 3b) in finer size steps than with the cyclone–filter setup. A 1.6 m-long stainless-steel probe with an inner diameter of 12 mm was inserted with its inlet at 90° to the flow direction in the flue gas channel and connected to the preheated (100 °C) DLPI. The DLPI sampled at a flow rate of ∼10 l/min, achieving the specified cutoff for each impactor stage. Impactor samples were typically collected for 15–30 s to avoid overloading of substrates and bounce-off effects. Samples for gravimetric analysis were collected using pregreased aluminum foils (Dekati, Tampere, Finland). Polycarbonate filters (Sartorius, Goettingen, Germany) were used for samples collected for chemical form analysis (sample details and results presented in ref. 17). The cutoffs of the stages (D_50_) in the DPLI are 23 nm, 46 nm, 85 nm, 150 nm, 240 nm, 390 nm, 640 nm, 1.0 μm, 1.7 μm, 2.6 μm, 4.2 μm, 7.0, and 10.7 μm. The cutoffs presented were corrected for the sampling temperature according to the impactor manufacturer’s procedure. To calculate the geometric mean diameter of the first impactor stage, an inlet cutoff of 15 μm was assumed for the sampling probe. This upper cutoff was estimated by considering the angle of sampling relative to the flow in the flue gas channel, the flue gas velocity, and the sampling flow rate. The samples for gravimetric analysis are denoted as Imp A, Imp B, and Imp C in Figure 2.

#### 2.2.3 Boiler and ESP Ash Samples

Boiler ash was collected from two positions in B2. Sample BoA_a was collected close to the furnace (at ∼500 °C), and BoA_b was collected from the other chutes (at ∼200 °C), as shown in Figure 1b. Boiler ash from B1 was, due to practical reasons, collected only at one position, shown in Figure 1a, corresponding to position “a” for B2 (closest to the boiler). The ESP sample was collected from B1, and the ESP was located directly after the aerosol sampling point (Figure 1a).

### 2.3 Online Measurements

The online instruments used for measuring the number size distributions of the fly ash were an aerodynamic particle sizer (APS 3321, TSI Inc.) and a scanning mobility particle sizer (SMPS 3936L10, including a condensation particle counter (CPC 3010), and a differential mobility analyzer (DMA 3081), TSI Inc.), as shown in Figure 3c. Combining the two instruments, a wide particle size range was covered, with the SMPS measuring the diameter size range of ∼20–700 nm, and the APS is used for larger, ∼0.7–10 μm diameter, particles. The SMPS classifies particles according to their electrical mobility diameter (*d*_me_), while the APS (as well as the impactor and cyclone) separates particles according to their aerodynamic equivalent diameter (*d*_ae_). The equations for diameter conversion are provided in Section 2.4.

With these instruments, the flue gas was sampled using a nonisokinetic dilution probe. The dilution probe consisted of two coaxial stainless-steel tubes. The dilution air was heated and supplied to the dilution point (close to the probe inlet) and passed through the cavity between the inner and outer tubes. Approximately 7 l/min of diluted gas was then extracted through the inner tube and further diluted in an ejector diluter (Dekati, Finland) positioned outside the flue gas channel. The dilution ratio of the respective dilution stages was about 1:10, resulting in an estimated total dilution ratio of 1:100. Using the first hot dilution stage, we avoided water vapor condensation. The second dilution stage was used to reduce the particle concentration to the measuring range of the aerosol instruments. The aerosol flow alternated between the APS and SMPS.

As the dilution system used has substantial losses of coarse particles, particularly in the ejector diluter, and as the APS does not fully cover the expected size range of the particles nor measure particles >10 μm accurately,^18^ no effort was made to perform the online measurements under isokinetic conditions. Although ejector diluters preserve the shape of the size distribution in the submicrometer range,^19^ turbulent flow causes losses of >1 μm-sized particles. For these reasons, only the SMPS data is shown in the Results section, and the APS data was only used to support the modal separation (size range of the minimum between the fine and coarse modes), which was also confirmed by the impactor samples.

### 2.4 Fitting Size Distributions and Converting between *d*_me_, *d*_ve_, and *d*_ae_

To visualize impactor distribution and extract the geometric mean diameter (GMD) and geometric standard deviation (GSD) of the different size distributions, data were fitted with a log-normal frequency function.^20^

Because different instruments and particle collection systems determine particle size using different measurement principles, they use slightly different definitions for the particle diameter. For example, the SMPS classifies particles according to their mobility diameter (*d*_me_), while the APS, impactor, and cyclone separate particles according to their aerodynamic diameter (*d*_ae_). The conversion between these size metrics is described by eq 1, based on the assumption of spherical particles.

1 $$d_{\mathrm{ae}}=d_{\mathrm{me}}\sqrt{\frac{\rho_{p}C_{c\left(d_{\mathrm{me}}\right)}}{\rho_{0}C_{c\left(d_{\mathrm{ae}}\right)}}}$$

where ρ_p_ is the particle density, ρ_0_ is the standard density (1 g/cm^3^), and *C*_c_ is the Cunningham slip correction factor, as defined in eq 2.

2 $$C_{c}\left(d\right)=1+\frac{2\lambda }{d}\left[\alpha +\beta \exp\left(\frac{\gamma }{\frac{2\lambda }{d}}\right)\right]$$

Here, *C*_c_(*d*) is the Cunningham factor for particles with diameter *d*, λ is the mean free path (66 nm in air at NTP^20^), and α, β, and γ are system-specific constants; for solid particles in air, α = 1.142, β = 0.558, and γ = 0.999.^21^

Because the impactor and SMPS (partially) cover the same particle size range, the particle density was estimated by comparing the number size distribution measured as a function of *d*_me_ with the SMPS to the mass size distribution obtained from the impactor (as a function of *d*_ae_). First, the normalized SMPS number distribution was converted to a volume size distribution under the assumption of a nonfractal particle morphology (i.e. spherical).^22^ The particle density (see eq. 1) was then adjusted so that the SMPS-derived volume size distribution matched the mass size distribution measured by the impactor. Only the shift in particle size of the distributions were used in the estimation, i.e., the normalized distribution and not the absolute masses. This was done because the absolute number concentrations reported by the SMPS are uncertain, as they are to a large degree affected by the uncertainties in the dilution factors and nonisokinetic sampling.

To plot the results from laser diffraction for coarse-mode particles in the same graph as the fine-mode distribution from impactor samples (see Section 2.5), *d*_ae_ was converted to the volume-equivalent diameter, *d*_ve_, using eq 3, assuming spherical particles.

3 $$d_{\mathrm{ve}}=d_{\mathrm{ae}}\sqrt{\frac{\rho_{0}\cdot C\left(d_{\mathrm{ae}}\right)}{\rho_{p}\cdot C\left(d_{\mathrm{ve}}\right)}}$$

### 2.5 Coarse Particle Size Distribution

All individual samples collected with the cyclone were pooled and analyzed with respect to particle size using laser diffraction. The ash samples were aerosolized in an RS01 capsule dry powder inhaler,^23^ and the size distribution was measured using a Malvern Spraytec instrument. A refractive index of 1.6 was used. All size distributions generated during a single measurement were averaged, and the measurement procedure was repeated six times. Laser diffraction measurements estimate the particle size in terms of *d*_ve_.

To plot the full size range of the mass distribution, from fine particles (measured with the impactor) to coarse-mode particles (measured by laser diffraction), the impactor data (sizing particles according to *d*_ae_) were converted to *d*_ve_ using eq 3, assuming a density of 2.8 g/cm^3^ (estimated as described in Section 2.4). The relative masses of the fine and coarse particle modes were scaled according to the gravimetric mass relation obtained from cyclone–filter sampling.

**Figure 4:**
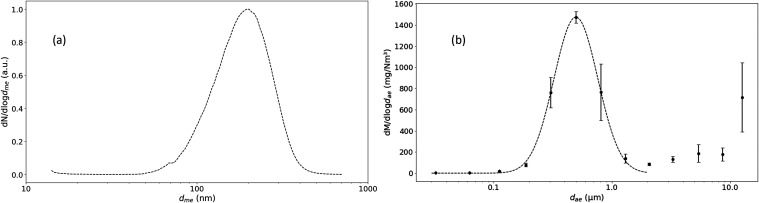
Particle size distribution in the flue gas channel. (a) Normalized number size distribution from SMPS measurements (average of nine scans). (b) Mass size distribution from the gravimetric analysis of impactor substrates (average of 3 measurements), together with the fitted log-normal distribution.

The laser diffraction size distribution for the coarse mode particles was fitted with two log-normal frequency functions.^20^ To enable comparision with the GMD of the fine particle mode obtained from the impactor measurements (reported as *d*_ae_), the resulting GMDs were converted to *d*_ae_ (using eq 3, assuming a density of 2.8 g/cm^3^) before being presented in the Result section. Note that this is not the same equivalent diameter shown in Figure 5 for the full range size distribution.

**Figure 5:**
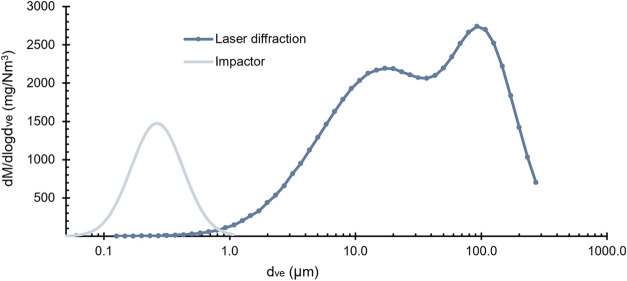
Mass size distribution from a combination of impactor data (<1 μm) and laser diffraction measurements after reaerosolizing coarse-mode particles. The relative intensity of the two modes is weighted according to the gravimetric analysis of the coarse and fine modes. Note that in the figure, the diameters are given as *d*_ve_, while the GMDs in the text correspond to *d*_ae_.

### 2.6 Elemental Analysis and Elemental Distribution in Fine and Coarse Ash Particles

All fly ash samples were analyzed for elemental content by an external accredited laboratory. Samples were digested with acid under heating and then analyzed in accordance with US EPA 200.8:1994 using inductively coupled plasma sector field mass spectrometry (ICP-SFMS). The elements included in the analysis were Be, Na, Mg, Al, Si, P, S, K, Ca, Sc, Ti, V, Cr, Mn, Fe, Co, Ni, Cu, Zn, As, Sr, Y, Zr, Nb, Mo, Cd, Sn, Sb, Ba, La, W, Hg, and Pb. All results are expressed as elemental mass per dry mass unit (mg/kg of dry mass).

For Cl analysis in samples from the cyclone-filter setup, the samples were first sintered at 550 °C with Na_2_CO_3_ and ZnO, water leached, and purified using cation exchange, and finally, the Cl content was determined using ICP-SFMS (US EPA 200.8). In the boiler and ESP ash samples, Cl was analyzed using ion chromatography after heated digestion in HNO_3_.

The partitioning of a given element in fine-mode particles was estimated based on the mass fraction in the respective particle mode on a dry basis, and the mass per dry weight of the given element according to eq 4.

4 $$X_{\mathrm{fine}}=\frac{c_{\mathrm{fine}}\cdot m_{\mathrm{fine}}}{c_{\mathrm{fine}}\cdot m_{\mathrm{fine}}+c_{\mathrm{coarse}}\cdot m_{\mathrm{coarse}}}$$

where *c*_fine_ is the concentration (mg/kg dry mass) of the element of interest (*X*) in the fine particle mode ash, *m*_fine_ is the fraction of (dry) mass in the fine mode, *c*_coarse_ is the concentration of the element in the coarse-mode ash (mg/kg dry mass), and *m*_coarse_ is the fraction of mass in the coarse mode. The element of interest in the coarse-mode particles was calculated as 1-*X*_fine_.

From a utilization and environmental safety perspective, it is relevant to compare the elemental content per dry mass unit in the respective ash fractions. Therefore, the ratio of elemental content in the fine fraction to the element content in the coarse fraction, i.e., *c*_fine_/*c*_coarse_, was calculated for each element and herafter referred to as F/C, and is shown in Table 2. This calculation was performed pairwise for the cyclone–filter samples collected in parallel, and the value presented is the average F/C. Elements with F/C ≫ 1 are enriched in fine particle fly ash, while F/C ≪ 1 indicates depletion. Note that this measure is different from *X*_fine_ (defined in eq 4), which is a more fundamental measure of elemental partitioning into fine and coarse particles.

## 3 Results and Discussion

### 3.1 Aerosol Particle Size Distributions

The number size distribution measured with the SMPS (20–700 nm) showed a distinct fine particle mode (Figure 4a), with a GMD of 190 nm and a GSD of 1.5. Due to substantial and size-dependent particle losses in the dilution system of particles larger than ∼4–5 μm, the APS data was ultimately not used (see Section 2.3). Nonetheless, the APS measurements supported the modal distribution, confirming that the fine (<1 μm) and coarse (>1 μm) particle modes were well separated (Figure S1) with a clear minimum in the distribution at ∼1.1 μm.

A clear separation between the fine and coarse particle modes was also apparent from the impactor size distribution, as shown in Figure 4b, with the mass distribution minimum at 1–3 μm. As for the online instruments, samples collected with the DLPI were strongly affected by nonisokinetic sampling, both due to the inlet orientation and preset flow rate for the DLPI (10 l/min). Based on the flow rates and angle of the sampling head, a rough estimation of the upper cutoff diameter for the sampling setup was made to be around 15 μm. However, an indication of the coarse mode could be captured using the impactor setup.

Fitting the mass size distribution from the impactor in the submicrometer size range with a log-normal distribution rendered a mass aerodynamic GMD of 0.50 μm and a GSD of 1.53, as shown in Figure 4b. The lower GMD obtained from the number size distribution (∼0.2 μm from the SMPS data) relative to the GMD of the mass size distribution is expected. This arises mainly from the inherent width of the distributions combined with the fact that mass scales with the cube of the particle diameter, resulting in mass-weighted metrics shifting toward larger particle sizes. The difference in the GMD is also affected by the shift arising from the conversion between the different equivalent diameters.

The mean mass concentration in the flue gas channel was determined to be 3.1 ± 0.5 g/Nm^3^, based on the sum of the cyclone–filter samples collected in parallel. On average, 16% of the total fly ash mass was present as fine-mode particles, and individual sample values are shown in Table 1. Both the impactor results and online instruments, as previously discussed, confirmed that the cyclone cutoff of 1.1 ± 0.1 μm (see Supporting Information) was suitable to separate fine and coarse-mode particles.

**Table 1:** Percent of Mass in Fine-Mode Particles^a^

Sample number	1	2	3	4	5
Percent of mass in fine-mode particles (_fine_)	17%	20%	13%	16%	13%

a The sample number corresponds to one fine and one coarse sample collected in parallel using the cyclone-filter setup. Data have been previously shown elesewhere.^17^

The size distribution of the coarse-mode particles was estimated from laser diffraction measurements of the particles collected in the cyclone. The laser diffraction volume size distribution in the range of 0.01 to 300 μm (*d*_ve_) is shown in Figure 5, along with the fine-mode mass distribution from the impactor. In the figure, the relative intensities of the fine and coarse modes from the respective instruments were scaled according to the average modal mass relation (Table 1).

According to the laser diffraction measurements, the coarse particles were composed of two modes with masses centered at 30 and 200 μm (*d*_ae_ from fit, details in Section 2.4), respectively, and again confirmed that the coarse mode was well separated from the mass peak in the fine mode, centered at ∼0.5 μm. In Figure 5, the distribution is cut at around 300 μm, while in some measurements, there is a third mode at even larger diameters (shown in Figure S3). As this mode was highly variable and the measurement uncertainty was known to be high for large diameter particles,^24^ the diameter is cut at 300 μm.

While the coarse mode accounts for most of the particle mass, converting the fitted mass size distribution to a number-based distribution shows that fine particles dominate by number, with only 0.1% of the particles in the coarse mode.

### 3.2 Particle Density

After converting the number size distribution, as measured by the SMPS, to volume-weighted size distribution, the size shift between the volume/mass size distributions as a function of *d*_me_ (SMPS data) and that as a function of *d*_ae_ (impactor data) was used to estimate the average particle density of the fine-mode particles (eqs 1, 2). The estimation was made assuming sphericity and constant density across the fine mode. The estimated density was 2.8 g/cm^3^ (Figure S2).

This estimation is in close agreement with the intrinsic density of MSW incineration fly ash previously reported as 2.78 g/cm^3^,^25^ while the same study suggested a lower effective density of 400 nm particles to be 2.32 g/cm^3^. The lower effective density of the 400 nm particles was explained by aggregated particles, which indicates that our assumption of close-to-spherical particles is reasonable. This is also close to the density expected if the fine-mode particles are dominated by alkali chlorides (2.0–2.7 g/cm^3^).

### 3.3 Elemental Composition

#### 3.3.1 Composition in Fine- and Coarse-Mode Particles

Fine and coarse particles had distinctly different elemental compositions, as shown in Table 2 and Figure 7 (individual sample values are presented in Table S2). Calcium, Si, and Al dominated the coarse particles by mass (not accounting for O). For fine-mode particles, Cl, Na, K, Zn, and S were the main constituents.

**Table 2:** Mean (SD) Elemental Composition in Units of mg/kg (Dry Weight) in Boiler Ash (BoA_a and BoA_b), ESP Ash, and Fine- and Coarse-Mode Particles Determined Using ICP-SFMS after Digestion (Details in Section 2.5)­^d^

	**BoA_a (B1 and B2)**	**BoA_b (B2)**	**ESP (B1)**	**Coarse (B1)**	**Fine (B1)**	**F/C** ^d^
**Al**	49,350 (6,604)	50,300 (1,200)	28,200	56,060 (11,120)	1,544 (833)	0.03
**As**	233 (165)	592 (107)	703	179 (61)	255 (124)	2
**Ba**	2,560 (894)	1,755 (95)	1,350	2,546 (552)	586 (444)	0.3
**Be**	1.8 (1.8)	0.9 (0.09)	0.5	1^a^	0.2^a^	-
**Ca**	233,500 (31,300)	205,500 (10,500)	147,000	260,800 (61,333)	8,157 (5,049)	0.03
**Cd**	20 (13)	69 (14)	328	57 (30)	836 (532)	**17**
**Cl**	12,181 (2,037)	24,907	94,189	44,389 (26,847)	334,970 (36,288)	**6**
**Co**	54 (22)	74 (2)	85	138 (90)	14 (6)	0.1
**Cr**	1,295 (390)	6,275 (2,705)	564	426 (124)	295 (392)	0.8
**Cu**	1,268 (691)	3,035 (575)	6,610	3,642 (1,496)	13,505 (9,247)	3
**Fe**	23,475 (9,155)	31,250 (8,050)	11,900	24,400 (2,611)	4,555 (1,880)	0.2
**Hg**	0.14 (0.05)	0.35 (0.07)	12	2 (2)	21 (32)^b^	**19**
**K**	17,925 (5,809)	24,250 (4,650)	68,600	17,060 (6,973)	126,029 (14,920)	**8**
**Mg**	19,325 (1,812)	24,000 (3,300)	11,900	22,440 (4,754)	775 (363)	0.04
**Mn**	1,018 (190)	1,025 (15)	588	1,310 (215)	234 (72)	0.2
**Mo**	84 (64)	115 (27)	106	59 (17)	54 (51)	0.8
**Na**	23,925 (4,715)	30,200 (5,400)	91,400	21,460 (6,443)	108,647 (28,043)	**6**
**Nb**	26 (20)	23 (4)	7	17 (2)	0.5 (0.3)	0.03
**Ni**	285 (207)	556 (61)	194	1,288 (1,268)	180 (169)	0.4
**P**	6,920 (1,317)	8,310 (170)	4,840	9,240 (1,886)	2,835 (1,706)	0.3
**Pb**	1,068 (1,200)	3,515 (675)	9,960	1,394 (634)	23,841 (26,141)	**17**
**S**	48,425 (17,545)	50,900 (900)	60,900	44,180 (11,177)	67,273 (23,459)	2
**Sb**	708 (210)	1,190 (50)	4,260	1,720 (673)	4,031 (1,761)	3
**Sc**	3 (2)	4 (0.1)	2	3 (1)	0.1^a^	0.03 ^a^
**Si**	101,425 (37,296)	83,550 (1,850)	58,000	74,860 (3,860)	25,814 (12,907)^c^	0.4
**Sn**	231 (88)	610 (10)	1920	453 (129)	2,529 (1,043)	**6**
**Sr**	-	-	-	608 (105)	54 (22)	0.09
**Ti**	15,750 (1,971)	15,050 (50)	7240	19,860 (4,535)	376 (144)	0.02
**V**	57 (13)	67 (6)	47	81 (16)	13 (8)	0.2
**W**	60 (45)	24 (6)	36	98 (64)	92 (154)	0.6
**Zn**	8,410 (2,422)	15,050 (750)	38,600	12,126 (3,976)	73,606 (20,885)	**6**
**Zr**	596 (903)	285 (51)	116	142 (32)	5 (5)	0.05

a Only one value was above the quantification limit.

b One value was below the quantification limit.

c Only analyzed in polycarbonate filters.

d F/C corresponds to the ratio of fine to coarse (Section 2.6); the ratio is marked in bold when it is higher than 5 and underscored when lower than 0.2.

On average, 81% of the fine-mode mass and 63% of the coarse-mode mass could be attributed to the elements that were analyzed. The elemental analysis does not include elements such as O, C, N, and H (see the list in Section 2.6). This is consistent with the observation that elements dominating the coarse mode (Ca, Si, Al, Fe, Ti, and Mg) are typically present in oxide-rich mineral phases with a higher proportion of oxygen compared to the salts dominating the fine mode, as described in a parallel study.^17^ That study, which focused on zinc speciation in the same samples as in the current study, presented X-ray diffraction data showing that the crystalline compounds of the coarse-mode particles were mainly CaSO_4_, CaCO_3_, Al_2_O_3_, Ca_2_Al_2_SiO_7_, SiO_2_, and Fe_2_O_3,_ whereas the fine mode was dominated by NaCl, KCl, (K,Na)_3_Na­(SO_4_)_2_, and K_2_ZnCl_4_, reflecting formation via evaporation–condensation processes.

To compare how each element partitions between fine- and coarse-mode particles, the relative mass fractionation of an element between the modes was calculated by combining the results from the gravimetric and elemental analyses, according to eq 4 (Section 2.6). The partitioning of each analyzed element in the respective mode is presented in Figure 6. More than half of the mass of Cd, Pb, K, Cl, Zn, Sn, and Na is present in fine-mode particles, in decreasing order, despite fine particles making up less than 20% of the total mass.

**Figure 6:**
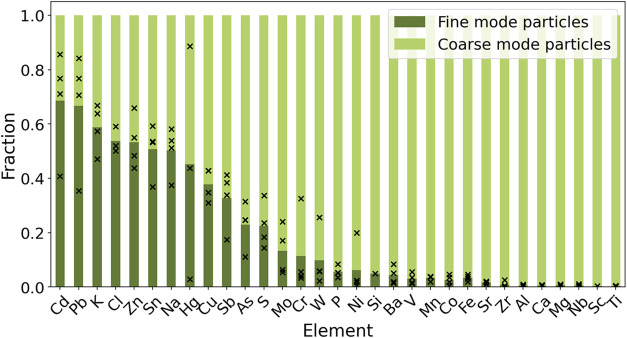
Partitioning of the analyzed elements between fine- and coarse-mode particles, calculated according to eq 4. The averages are shown as bars, and individual measurements as crosses. Each cross corresponds to one pair of fine- and coarse-mode samples collected in parallel (cyclone and filter coupled in series).

Cadmium, K, Cl, Zn, and Na compounds typically have high vapor pressures, explaining their partitioning in the fine mode formed by evaporation–condensation, although their volatility is strongly dependent on the chemical form. Although the volatility of elemental Pb is low, enrichment in fine particles could be explained by more volatile Pb forms, such as halides. For example, high levels of Cl in the fuel may result in the volatile combustion product PbCl_2_.^26,27^ Elemental Hg and Hg compounds are typically volatile. Our samples showed substantial variability between fine- and coarse-mode particle fractions, with low concentrations in both modes across all samples, except for one sample (Fine5/Coarse5), which also exhibited the strongest tendency for Hg to partition into fine particles.

Although ESPs efficiently remove most heavy metals, it is known that Hg is not always captured from the flue gas by the ESP but is removed later in the flue gas cleaning system. Efficient capture requires Hg adsorption on surfaces (fly ash particles), which is more likely for oxidized Hg.^28^ As Hg oxidation in flue gas is kinetically limited,^28^ its behavior will also depend on many process parameters such as the fuel composition, fly ash concentration, and flue gas cooling rate, which makes it difficult to predict whether it will remain in the gas phase.

Typical refractory elements (e.g., Ca, Al, Mg, Ti, Nb, and Zr) were found almost exclusively in coarse-mode particles, as expected for particles originating from entrainment in the combustion zone.

Previously, Zeuthen and colleagues^15^ used a low-pressure impactor to collect fine (<2 μm) particles in a grate-fired MSW boiler and compared the elemental characteristics of this ash fraction to the full ash collected in large scale bag-house filters. Except for Sn, which was not included in the analysis, the same elements (Cd, Pb, K, Cl, Zn, Na) as in the present study were found to be enriched in the smallest fly ash particles. In another study on grate-fired MSW incineration, Brunner and colleagues^13^ used a high-temperature impactor to sample particles <3 μm at different positions in the flue gas channel (different temperatures). Furthest away from the furnace, at a position with a relatively similar temperature (350 °C) to our sampling point, Cl, Na, K, Zn, and S dominated across fine particle sizes, which is also in agreement with our findings. To the best of our knowledge, this study is the first to demonstrate the effective separation of coarse aerosol samples from fine fly ash particles.

From a circular-economy perspective, in relation to cost-effective material recycling or secondary use of ash from waste incineration for construction, the mass of each element per total mass in the respective ash type is a key metric, as presented in Table 2. In the table, elemental enrichment in fine vs coarse-mode ash is indicated as F/C. F/C is defined as *c*_fine_ (mg/kg dry mass) divided by *c*_coarse_.

Elements that are enriched in fine particle ash compared with the coarse fraction (and ESP ash) include those that are both ecotoxic and/or potentially valuable for resource recovery. For example, Cd and Pb show concentrations approximately 17-fold higher in the fine-mode ash, Zn and Sn about 6-fold higher, Cu and Sb about 3-fold higher, and As about 2-fold higher.

#### 3.3.2 Composition Boiler and ESP Ash

The elemental compositions of bulk fly ash samples from the ESP and boiler are presented in Table 2 and in Figure 7. For comparison, the content of the aerosol samples (fine- and coarse-mode particles) is shown in Figure 7. The elemental content of the individual boiler samples is presented in SI, Table S2. The category “Other” (Figure 7) represents uncharacterized elements (e.g., O and C). The “Other” category makes up 34% of the mass in the ESP sample and between 40 and 44% in the boiler ash samples.

**Figure 7:**
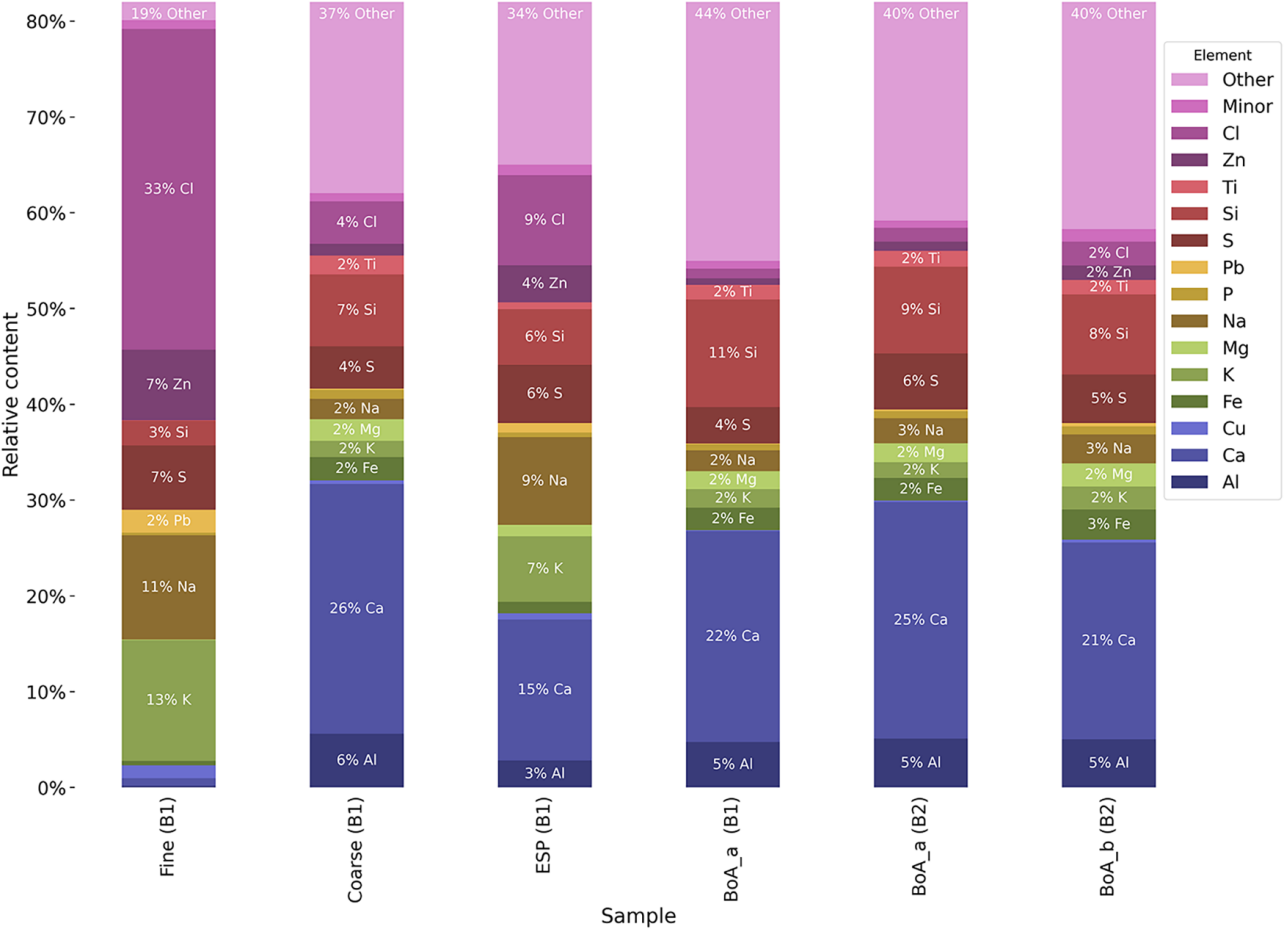
Average relative elemental composition of ESP and boiler ash samples from elemental analysis. The minor category represents the sum of the analyzed elements, which make up less than 1% of the mass. The “Other” category is likely uncharacterized elements such as O, C, and H. Note that the vertical axis is cut at around 80%.

The elemental composition of the boiler ash samples resembles that of coarse-mode particles, with large mass fractions of Ca, Si, Al, and S. At B1, the WtE unit where aerosol samples were collected, boiler ash could only be collected at one position, which was relatively close to the furnace. To investigate the deposits in different regions in the flue gas channel, boiler ash was also sampled from a similar WtE unit, B2, placed at the same facility and incinerating the same waste stock, allowing samples to be collected at one additional position further down the boiler (Figure 1). Due to the temperature difference between sampling points *a*, where the flue gas is at ∼800 to 500 °C, and *b*, where the temperature is ∼200 °C, the boiler ash sampled at position *b* was expected to contain more condensates.

Boiler ash collected at position *b* had higher contents of Cl, Cd, Cr, Cu, Pb, Sn, and Zn, while ash collected at position *a* had slightly higher contents of Be than *b* (Tables 2 and S2). With the exception of Cr, these trends are in line with those found in the fine-coarse fraction comparison, suggesting that the increase of these elements in boiler deposits at position *b* is due to condensation. Chromium concentrations were higher in the boiler ash sampled at position *b* than at *a.* No corresponding increasing trend in Cr concentration was found in the fine fraction mode. However, the variation in the concentration between the boiler ash samples from the same position and WtE unit was considerable (Table S2), and additional samples are needed for more reliable quantification. On average, the boiler ashes from the two units, B1 and B2, were similar in composition.

The elemental composition of the ESP ash (Figure 7 and Table 2) resembled a mixture of fine (16% of total mass) and coarse-mode particles (84% of total mass), in line with what would be expected for efficient ESP collection of both fine and coarse particles from the flue gas.^29^ Compared to the boiler ash samples, ESP ash contained more K, Na, and Zn and less Ca and Al.

## 4 Implications for Secondary Use

Our results support that the separation of fine and coarse particles, if implemented at a large scale, could in the future contribute to optimizing the circular use of fly ash from both an environmental safety perspective and a metal recovery standpoint.

Coarse particles and boiler ash are enriched in Fe, Al, Si, Ca, and Mg (Table 2), which are essential elements for many construction materials, such as cement.^30^ This suggests that the coarse-mode fly ash fraction may have suitable properties for construction applications.^31^ Moreover, separating and utilizing the coarse ash fraction after removing the fine fraction would substantially reduce the concentrations of Cd, Pb, Hg, As, Cu, and Zn compared to the bulk fly ash collected in ESP filters. Consequently, the ecotoxicity of the coarse fraction is expected to be markedly lower than that of the fine fraction, as well as that of the ESP ash, which is commonly classified as hazardous waste.^32^ In addition to lower concentrations of several potentially toxic elements, there are indications that the chemical forms present in coarse-mode particles are more stable in terms of solubility than those in the fine fraction. This inference follows from the contrasting formation mechanisms of the two particle modes and is further supported by XRD and Zn-XAS results.^17^

Contrary to the coarse fraction, the fine particles were enriched in metals of industrial value (e.g., Zn, Cd, Cu, Pb) and could be targeted for more efficient recovery of metals along with alkali salts (K, Na, and Cl). In particular, if the metal of interest occurs in more soluble forms in fine mode ash, as demonstrated for Zn.^17^

The realizability of industrial-scale size separation remains to be investigated; however, approaches such as multicyclones or pencil-type cyclones for the collection of coarse fly ash particles are traditionally used at an industrial scale. The main challenge lies in reaching the low cutoff suggested here (1 μm) for the optimal separation of the two modes in industrial-scale flue gas cleaning systems. An approach to solve this could be combining the fine-tuning of the boiler operation, e.g., combustion air distribution, and the ash abatement design. The placement of the separators along the flue gas channel should also be critically evaluated in relation to surface condensation and operational efficiency. However, we want to stress that any separation of the coarse and fine modes, even with a higher cutoff than that for optimal modal separation, could still be useful.

## Supplementary Material



## Notes

Description of testing cyclone cutoff in the lab; mass size distribution measured with APS (Figure S1); results from cyclone cutoff measurements (Figure S2); mass size distributions from SMPS and DLPI for density estimation (Figure S3); laser diffraction mass size distribution (Figure S4); aerosol mass concentration of elements (Figure S5); elemental composition in individual fine and coarse samples (Table S1); elemental composition in individual boiler samples (Table S2) (PDF)
