# Supplementary material for: Physicochemical Properties of Fine and Coarse Fly Ash Aerosol Particles from Waste Incineration
Source: ACS Environ Au. 2026 Jun 2;6(4):654–64. doi: 10.1021/acsenvironau.6c00018 (PMC13377509; doi:10.1021/acsenvironau.6c00018)
Supplement: Supplementary file 1 [file vg6c00018_si_001.pdf]

Supporting Information - Physicochemical properties of fine and coarse fly ash aerosol particles from waste incineration

Fanny Bergman[1, 2], Jenny Rissler[*1,2], Sara Janhäll[3], Michael Strand[4], Edvin Elmroth[1,5], Karin Karlfeldt-Fedje[6,7]

[1] *Ergonomics and Aerosol Technology, Lund University, Lund, 221 00, Sweden*

[2] *NanoLund, Lund University, Lund, 221 00, Sweden*

[3] *RISE Research Institutes of Sweden, Borås, 501 15, Sweden*

[4] *Built Environment and Energy Technology, Linnaeus University, Växjö, 351 95, Sweden*

[5] *NG Nordic AB, Kumla, 692 92, Sweden*

[6] *Renova Recycling and Waste Management, Renova AB, Gothenburg, 401 22, Sweden*

[7] *Architecture and Civil Engineering, Chalmers University of Technology, Gothenburg, 412 96, Sweden*

**Corresponding author*

Supplementary figure

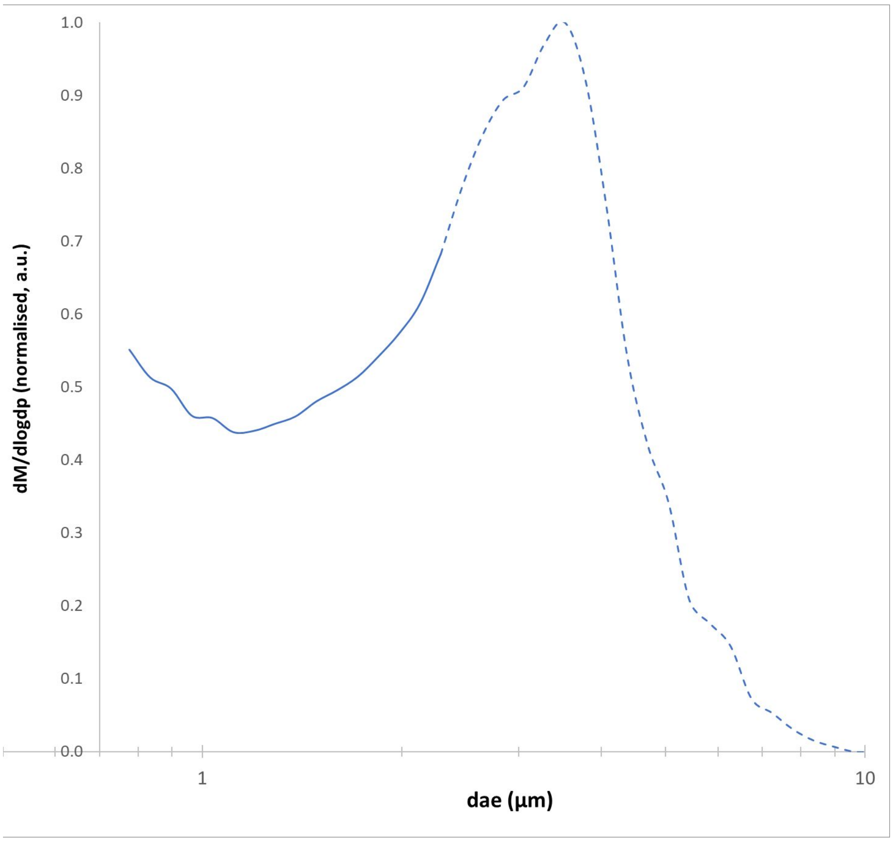

Figure S1. Normalized APS mass size distribution in flue gas channel.

Determining cyclone cut-off

A sharp cut-off aluminum cyclone with body diameter 35 mm was used for coarse particle collection. The cut-off diameter (D_{50}) of the cyclone was determined experimentally and compared with calculated values,[1] Figure S1. Values were calculated using a body diameter of 35 mm (as specified for the cyclone used) for comparison with experimental data. For experimental determination, ammonium sulphate (1 M) was nebulized, passed through a diffusion drier and sampled using APS 3321. A vacuum pump was used to achieve the desired flow rates, from 10 to 35 l/min. Measurements were performed alternating between measuring with and without cyclone, with at least three repetitions per flow rate.

The size distribution measured with cyclone was divided by the size distribution measured without cyclone and the cut-off, D_{50}, was determined from where this quotient was 50%. Due to pressure differences when connecting the cyclone at flow rates of 25 l/min and higher, APS size distributions and thus cut-off estimates are more uncertain for high flow rates (shown as black circles). Flow rates in the field study were 36 ± 3 l/min (mean ± SD), and the cut-off estimated to be 1.1 ± 0.1 µm.

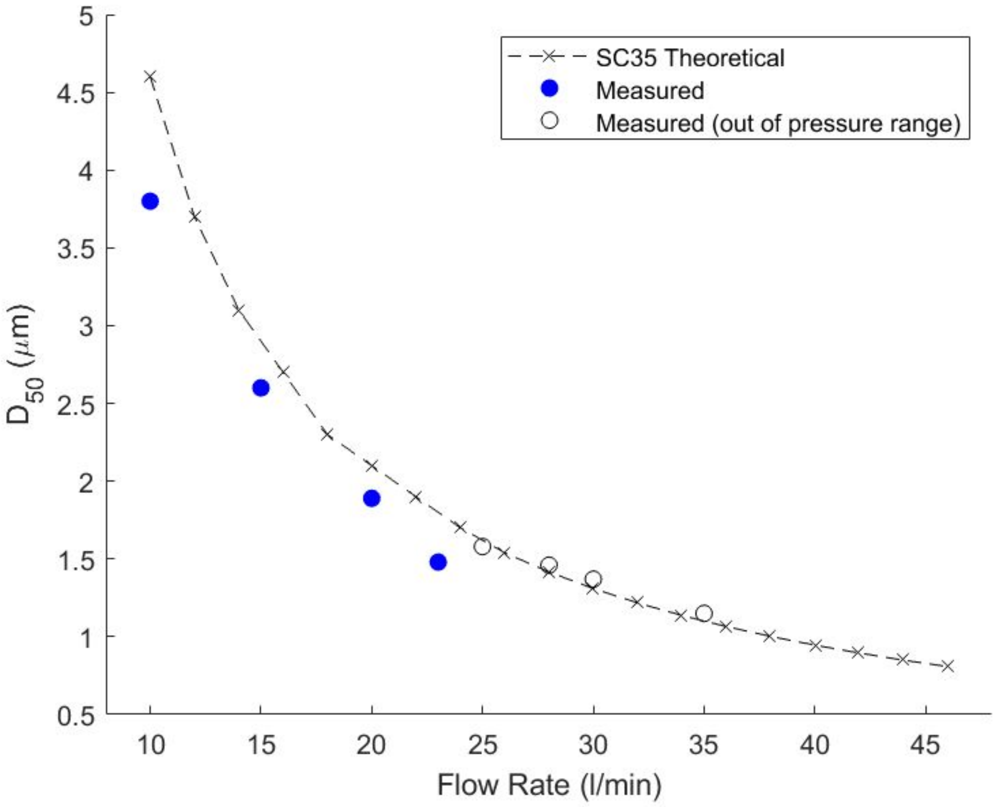

Figure S2. Cyclone cut-off diameter estimated experimentally at flow rates ranging from 10 to 35 l/min (blue circles) in relation to cut-offs calculated from cyclone body diameter 35 mm and type (sharp cut-off).

Supplementary figures

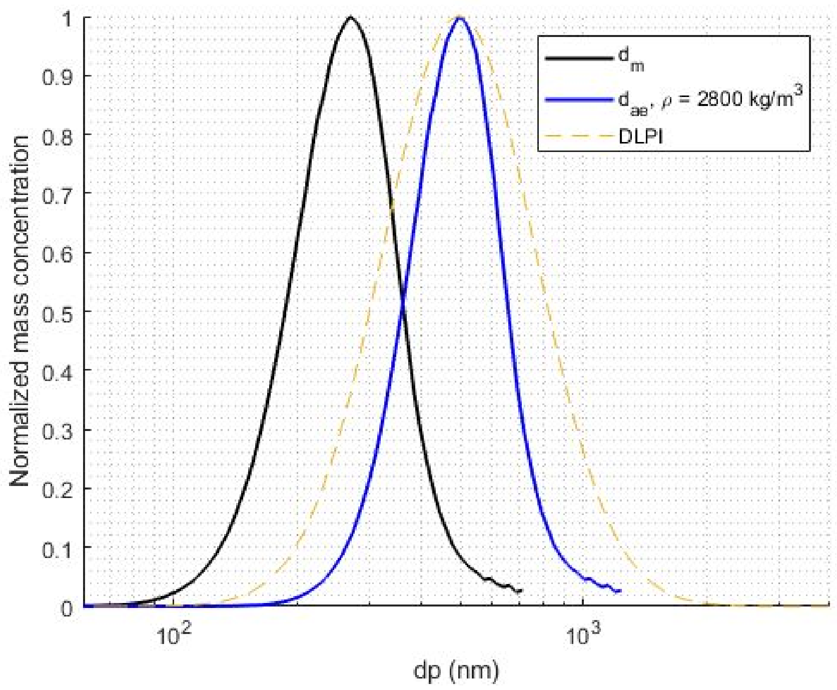

Figure S3. Particle density estimation from impactor (DLPI, d_{ae}) fit and SMPS measurements, used converting mobility (d_m) to aerodynamic equivalent diameter (d_{ae}) by applying a specific particle density.

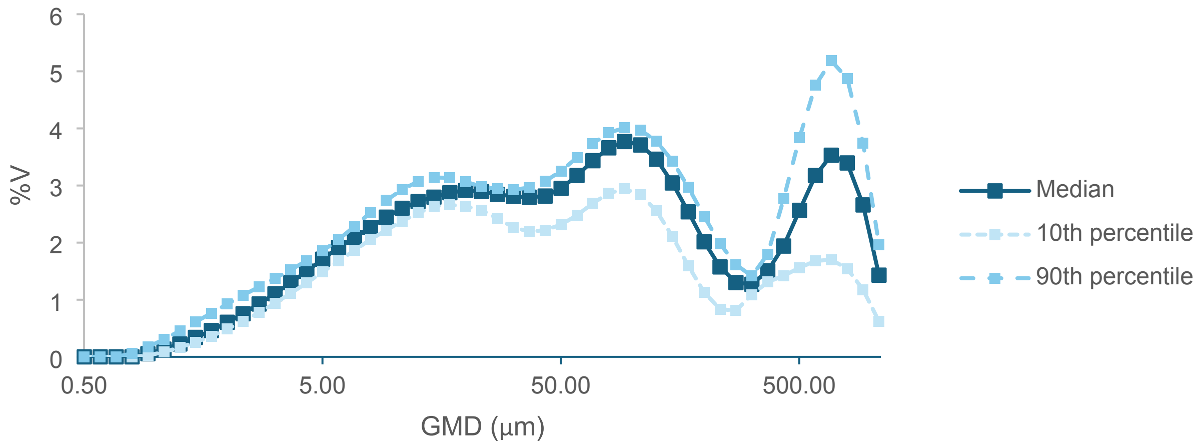

Figure S4. Laser diffraction particle size distribution.

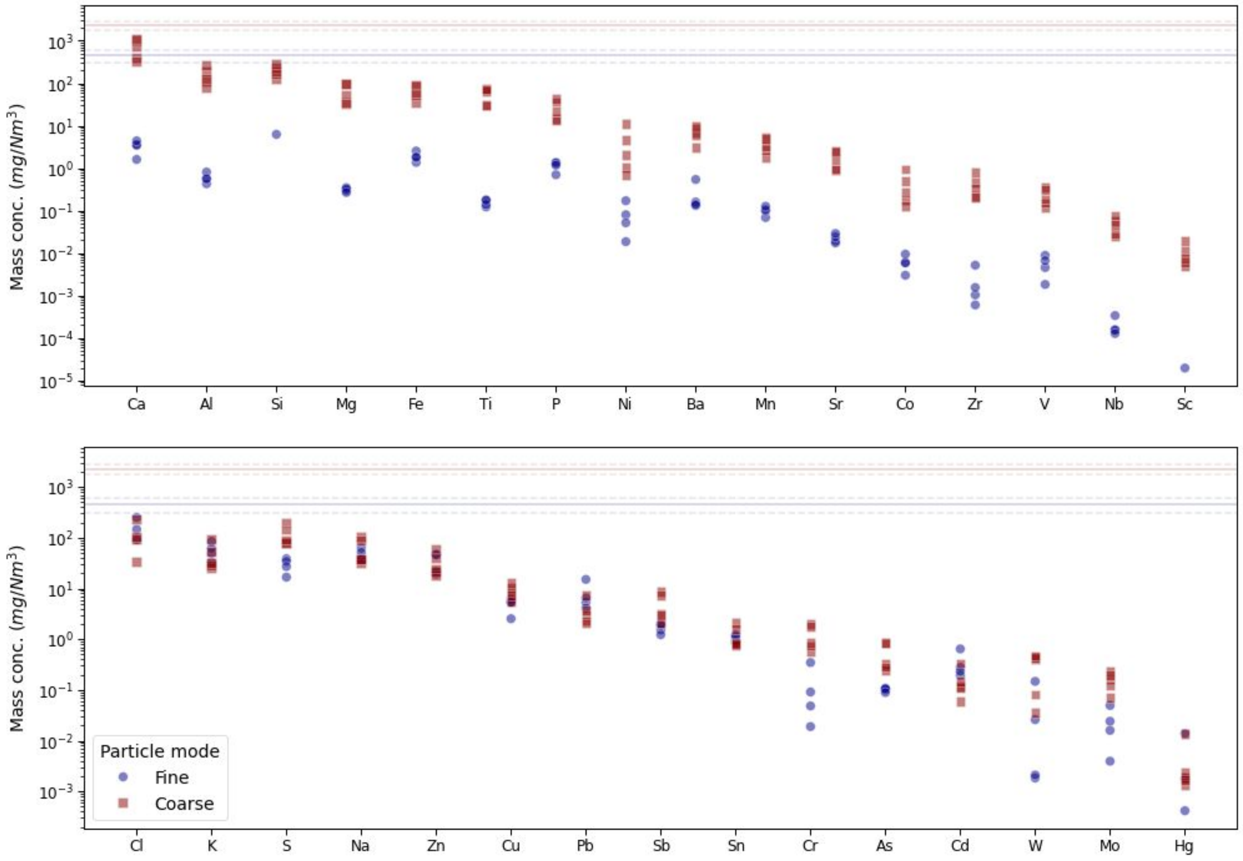

Figure S5. Mass concentration per air volume of individual elements in fine (blue dots) and coarse (red squares) mode particles. The total mass concentration in respective mode is given as a solid (mean) and dashed (standard deviation) line.

Supplementary tables

Table S1. Elemental composition in fine and coarse mode particles determined with ICP-SFMS after digestion (details in section 2.5). Fine1 was collected simultaneously with Coarse1 and likewise Fine2 and Coarse2 etc.

	Coarse (mg/kg)					Fine (mg/kg)			
	Coarse1	Coarse2	Coarse3	Coarse4	Coarse5	Fine1	Fine2	Fine4	Fine5
**Al**	38 900	59 300	65 900	51 600	64 600	2 057	529	1 220	2 370
**As**	123	259	212	113	190	276	131	194	418
**Ba**	3 350	2 740	2 350	2 440	1 850	335	171	1179	660
**Be**	<LOQ	1	<LOQ	<LOQ	<LOQ	<LOQ	<LOQ	<LOQ	0.2
**Ca**	166 000	329 000	280 000	287 000	242 000	4 066	4 291	9 572	14 698
**Cd**	30	104	37	45	70	492	287	1 406	1 158
**Cl**	NA	71 081	8 235	42 121	56 119	NA	308 235	320 396	376 279
**Co**	64	295	124	106	103	15	7	21	12
**Cr**	375	634	445	342	336	882	113	106	79
**Cu**	5 230	1 750	3 160	2 960	5 110	13 618	3 141	11 683	25 580
**Fe**	26 500	27 300	23 200	24 200	20 800	6 408	2 182	3 979	5 651
**Hg**	1	4	0.4	0.5	1	5	0.5	<LOQ	58
**K**	14 600	29 400	13 200	12 800	15 300	125 867	105 059	135 050	138 140
**Mg**	17 200	29 400	24 500	21 800	19 300	886	401	583	1 230
**Mn**	1 340	1 640	1 220	1 300	1 050	247	129	276	284
**Mo**	81	72	49	49	42	126	20	53	16
**Na**	19 400	32 300	21 600	15 500	18 500	99 520	77 624	112 792	144 651
**Nb**	15	18	19	19	15	0.9	0.2	0.3	0.7
**Ni**	356	3 500	1 120	833	631	434	99	112	77
**P**	6 560	11 200	10 900	8 830	8 710	1 778	1 682	2 554	5 326
**Pb**	1 150	2 320	920	819	1 760	13 507	5 092	14 209	62 558
**S**	39 700	62 800	35 800	36 500	46 100	98 311	42 471	59 010	69 302
**Sb**	1 030	2 730	1 800	1 180	1 860	3 128	2 293	4 354	6 348
**Sc**	3	4	5	3	3	<LOQ	<LOQ	<LOQ	0.1
**Si**	81 300	73 100	71 500	75 400	73 000	NA	NA	NA	25 814
**Sn**	386	648	390	325	514	2 148	1 516	2 480	3 973
**Sr**	472	754	631	633	552	44	30	63	80
**Ti**	15 000	22 800	17 800	26 200	17 500	437	172	388	505
**V**	76	109	75	75	68	22	6	14	8
**W**	18	128	116	177	49	5	32	322	8
**Zn**	9 280	18 800	9 840	9 910	12 800	55 289	58 706	100 198	80 233
**Zr**	103	146	192	137	132	13	1	3	4

Table S2. Boiler ash elemental composition in mg/kg dry mass. * denotes Cl samples that were not analyzed individually but for weighted mixes of BoA_a1 (B1) + BoA_a2 (B1), BoA_a1 (B2) + BoA_a2 (B2), and BoA_b1 (B2) + BoA_b2 (B2), respectively.

Sample/ Element	BoA_a1 (B1)	BoA_a2 (B1)	BoA_a1 (B2)	BoA_a2 (B2)	BoA_b1 (B2)	BoA_b2 (B2)
Al	42 900	52 300	45 000	57 200	51 500	49 100
As	451	76	264	140	698	485
Ba	1 630	3 340	1 960	3 310	1 660	1 850
Be	0.7	1.0	0.9	4.5	1.0	0.8
Ca	244 000	196 000	224 000	270 000	195 000	216 000
Cl	10 144*	10 144*	14 218*	14 218*	24 907*	24 907*
Cd	21	8	37	15	83	56
Co	31	54	48	85	72	76
Cr	1 640	1 600	841	1 100	8 980	3 570
Cu	1 640	545	2 040	847	2 460	3 610
Fe	12 400	34 500	21 600	25 400	39 300	23 200
Hg	0.1	0.1	0.2	0.2	0.4	0.3
K	24 600	13 900	20 900	12 300	28 900	19 600
Mg	19 500	18 300	17 700	21 800	27 300	20 700
Mn	791	1 080	959	1 240	1 040	1 010
Mo	59	38	61	180	142	88
Na	24 300	19 300	30 300	21 800	35 600	24 800
Nb	12	21	15	56	27	19
Ni	104	189	268	579	617	495
P	7 760	5 040	6 980	7 900	8 140	8 480
Pb	847	183	2 820	421	4 190	2 840
S	41 100	35 000	74 200	43 400	50 000	51 800
Sb	486	581	934	832	1 140	1 240
Sc	1	7	4	2	4	3
Si	69 800	155 000	84 900	96 000	85 400	81 700
Sn	176	143	336	268	619	600
Se	566	526	518	652	449	508
Ti	14 200	16 100	14 300	18 400	15 000	15 100
V	42	73	52	63	73	60
W	17	89	27	107	18	29
Y	16	21	13	13	13	13
Zn	6 300	7 630	11 900	7 810	15 800	14 300
Zr	112	1950	152	169	336	234
La	14	23	19	20	23	24
Li	23	17	35	37	55	54

References

(1) Kenny, L. C.; Gussman, R. A. A DIRECT APPROACH TO THE DESIGN OF CYCLONES FOR AEROSOL-MONITORING APPLICATIONS. *Journal of Aerosol Science* **2000**, *31* (12), 1407–1420. DOI: https://doi.org/10.1016/S0021-8502(00)00047-1.
